# Direct UAV-Based Detection of *Botrytis cinerea* in Vineyards Using Chlorophyll-Absorption Indices and YOLO Deep Learning

**DOI:** 10.3390/s26020374

**Published:** 2026-01-06

**Authors:** Guillem Montalban-Faet, Enrique Pérez-Mateo, Rafael Fayos-Jordan, Pablo Benlloch-Caballero, Aleksandr Lada, Jaume Segura-Garcia, Miguel Garcia-Pineda

**Affiliations:** 1 Computer Science Department, ETSE—Universitat de València, 46100 Valencia, Spain; guillem.montalban@uv.es (G.M.-F.); enpema2@alumni.uv.es (E.P.-M.); rafael.fayos@uv.es (R.F.-J.); pablo.benlloch@uv.es (P.B.-C.); 2 Knowledge Genesis Group, Smart Enterprise, Samara State Technical University, 443100 Samara, Russia; ladalexer@gmail.com

**Keywords:** remote sensing, UAV, multispectral, CNN, YOLO, AI, Agriculture 5.0

## Abstract

The transition toward Agriculture 5.0 requires intelligent and autonomous monitoring systems capable of providing early, accurate, and scalable crop health assessment. This study presents the design and field evaluation of an artificial intelligence (AI)–based unmanned aerial vehicle (UAV) system for the detection of *Botrytis cinerea* in vineyards using multispectral imagery and deep learning. The proposed system integrates calibrated multispectral data with vegetation indices and a YOLOv8 object detection model to enable automated, geolocated disease detection. Experimental results obtained under real vineyard conditions show that training the model using the Chlorophyll Absorption Ratio Index (CARI) significantly improves detection performance compared to RGB imagery, achieving a precision of 92.6%, a recall of 89.6%, an F1-score of 91.1%, and a mean Average Precision (mAP@50) of 93.9%. In contrast, the RGB-based configuration yielded an F1-score of 68.1% and an mAP@50 of 68.5%. The system achieved an average inference time below 50 ms per image, supporting near real-time UAV operation. These results demonstrate that physiologically informed spectral feature selection substantially enhances early *Botrytis cinerea* detection and confirm the suitability of the proposed UAV–AI framework for precision viticulture within the Agriculture 5.0 paradigm.

## 1. Introduction and Related Work

**Figure 1:**
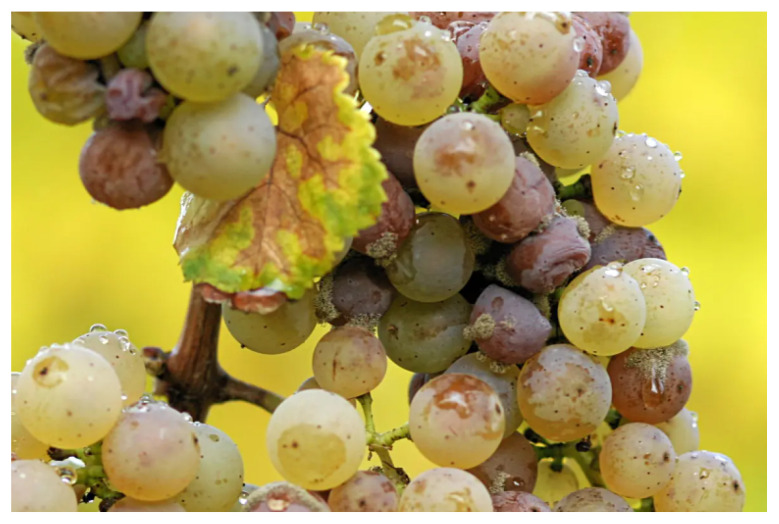
Grapes affected by *Botrytis cinerea* in a vine.

Early and reliable detection of plant diseases remains a central challenge in precision agriculture, particularly when symptoms are subtle and spatially heterogeneous. From a sensing perspective, the key difficulty lies in capturing early physiological changes in vegetation that precede visible damage and translating these signals into actionable information at field scale. In viticulture, fungal pathogens such as *Botrytis cinerea* induce biochemical and structural alterations—most notably chlorophyll degradation and changes in leaf optical properties—that can be detected through appropriately designed remote sensing systems. Figure 1 shows a bunch of grapes on a vine affected by Botrytis fungus.

Unmanned aerial vehicles (UAVs) equipped with multispectral sensors have emerged as an effective platform for high-resolution, site-specific crop monitoring. Compared with satellite-based observations, UAVs offer finer spatial resolution, flexible acquisition timing, and controlled viewing geometry, making them particularly suitable for detecting localized stress patterns in vineyards. Multispectral imaging systems capturing reflectance in the visible, red-edge, and near-infrared (NIR) bands enable the computation of vegetation indices that are sensitive to chlorophyll concentration, pigment composition, and canopy condition. Numerous studies have demonstrated that such indices can reveal plant stress before visual symptoms become apparent, provided that radiometric calibration and band alignment are properly addressed [1,2,3].

While traditional vegetation indices such as NDVI are widely used to assess vegetation vigor, they are strongly influenced by canopy structure, soil background, and illumination conditions, which can limit their sensitivity to early-stage disease. In contrast, indices derived from chlorophyll absorption features—particularly those exploiting red and red-edge reflectance—have been shown to provide greater physiological specificity. Indices such as the Chlorophyll Absorption Ratio Index (CARI) and its variants were originally developed to isolate chlorophyll absorption effects while reducing background and brightness influences, making them well suited for detecting subtle pigment-related changes associated with biotic stress [4,5]. UAV-based studies have confirmed that red-edge and chlorophyll-sensitive indices improve discrimination of early stress compared with RGB or broadband indices alone [6,7].

Transforming multispectral observations into reliable disease detection outputs increasingly relies on machine learning and deep learning techniques. Convolutional neural networks (CNNs) have become the dominant approach for extracting discriminative spatial–spectral features from aerial imagery, outperforming classical pixel-based classifiers in robustness and scalability. Among these methods, real-time object detection architectures such as You Only Look Once (YOLO) are particularly attractive for UAV applications, as they provide a direct mapping from sensor data to spatially localized detections with low inference latency [8,9]. Recent adaptations of YOLO models for agricultural sensing have demonstrated their effectiveness in detecting small objects and localized anomalies under operational conditions [10,11].

Despite these advances, most existing UAV-based approaches to vineyard disease monitoring either rely on RGB imagery, perform pixel-level semantic segmentation, or focus on risk estimation rather than direct pathogen detection. Studies addressing *Botrytis cinerea* specifically are relatively scarce and often produce probabilistic susceptibility maps rather than explicit, geolocated detections of infected plant regions. Moreover, the integration of calibrated multispectral vegetation indices as direct inputs to real-time object detection networks remains limited, particularly for chlorophyll-absorption–based indices that are physiologically linked to early disease progression.

Recent surveys consolidate how UAV sensing and AI have become the dominant pipeline for field-scale crop health monitoring [5,12]. Multispectral and hyperspectral payloads on small UAVs are now standard for early stress and disease detection, with systematic reviews reporting rapid growth in publications, common sensor–altitude configurations, and typical accuracy ranges across crops and pathosystems. These works also emphasize persistent gaps in domain transfer, robustness under variable illumination, and the lack of standardized benchmarks for reproducible evaluation [12,13].

Deep-learning methods have clearly surpassed traditional image-processing pipelines. Surveys consistently show that CNN-based architectures outperform classical approaches for agricultural computer vision tasks, making models such as YOLO and RetinaNet the de facto standards for real-time detection in UAV imagery. Recent advances adapt YOLO variants to agronomic constraints—small objects, occlusion, and inter-class similarity—achieving substantial gains through anchor refinement, multiscale feature fusion, and attention mechanisms [9,10,11]. Spectral information remains equally important: red-edge and near-infrared–based vegetation indices (e.g., CARI/MCARI, CIRE) have proven particularly effective for early stress detection, with UAV studies reporting higher precision and recall when VI layers are fused with RGB or used directly as network input [5,12].

Within this broader research landscape, fungal diseases represent a major challenge, especially those with subtle early symptoms. *Botrytis cinerea* is one of the most impactful pathogens in grapevine cultivation, affecting multiple plant organs and causing significant yield and quality losses. Despite the rapid expansion of UAV- and AI-based vineyard monitoring, dedicated approaches for direct detection of *B. cinerea* remain scarce.

Three recent works illustrate the current state and limitations of related methods. Pinheiro et al. [6] developed a YOLO-based system to detect grape clusters and classify them as healthy or damaged based on visible lesions. Using RGB images captured with a handheld smartphone and manually annotated datasets, they evaluated several YOLO variants (YOLOv5x6, YOLOv7-E6E and YOLOR-CSP-X), reporting an F1-score of up to 94% for the detection of damaged clusters. Although promising, the approach is restricted to RGB imagery, limiting early symptom detection, and does not target specific pathogens such as *B. cinerea*.

Kerkech et al. [4] proposed a multispectral UAV-based system to detect downy mildew through semantic segmentation. They introduced a non-rigid AKAZE-based registration method to align visible and infrared bands, followed by SegNet to classify pixels into soil, shadow, healthy vegetation, or symptomatic vegetation. The method achieved 92% plant-level accuracy in two real vineyards, demonstrating the value of multispectral sensing. However, its semantic segmentation approach is less suitable for object-level detection or geolocated infection mapping, and it targets downy mildew rather than *B. cinerea*.

Vélez et al. [7] presented a methodology to estimate *B. cinerea* risk using multispectral UAV imagery and vegetation and structural predictors (NDVI, LAI, DTM, CHM). A Random Forest classifier trained on 153 georeferenced vines achieved an $R^{2}$ close to 0.7, with LAI being the most informative variable. The study produced spatial risk maps through kernel density estimation, showing a higher susceptibility in dense-canopy areas at low altitude. Although valuable for risk assessment, the approach does not employ deep learning and does not perform direct pathogen detection.

Overall, the current literature demonstrates strong progress in UAV-based disease monitoring, but significant gaps remain: (i) limited integration of calibrated multispectral indices with advanced deep-learning detectors, (ii) a scarcity of object-detection approaches specifically targeting *B. cinerea*, and (iii) the lack of methods capable of producing directly geolocatable infection hotspots for precision agriculture operations.

The current study addresses these gaps by combining calibrated multispectral orthophotos, vegetation index computation, and a YOLO-based object detection model specifically trained for *Botrytis cinerea*. The resulting system provides a direct, automatic and spatially explicit detection method aligned with the requirements of modern precision viticulture and the broader vision of autonomous Agriculture 5.0. Table 1 summarizes the main key points of each contribution with respect to our contribution. To the best of our knowledge, no previous study combines calibrated multispectral vegetation indices with real-time object detection to directly localize *Botrytis cinerea* infections from UAV imagery.

**Figure 2:**
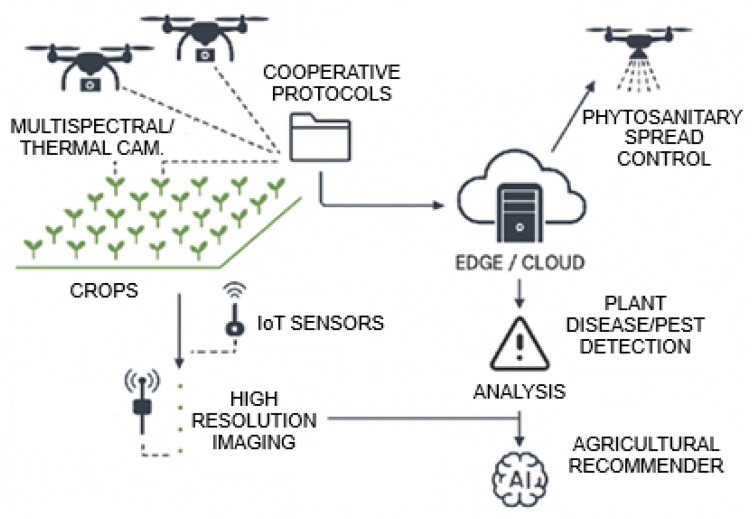
Architecture of technological systems with application in Agriculture 5.0.

To summarize, in this work we address the limitations found in previous works by presenting a UAV-based sensing and detection framework for the direct identification of *Botrytis cinerea* symptoms in vineyards. The proposed system integrates radiometrically calibrated multispectral UAV imagery with chlorophyll-sensitive vegetation indices and a YOLOv8 object detection model. Figure 2 presents a summary of this description of the combination of technologies to help the agricultural sector advance in the battle against potential problems in agriculture. By systematically comparing RGB imagery with chlorophyll-absorption–based indices under identical detection architectures, the study evaluates the contribution of physiologically informed spectral features to detection accuracy. The resulting framework produces spatially explicit, geolocated detections with near real-time inference capability, aligning with the requirements of precision viticulture and sensor-driven crop monitoring.

The main contributions in this paper are related to the specification of a UAV-based object-detection framework specifically trained for direct detection of *Botrytis cinerea* symptoms (here we avoid a risk estimation for the disease). Also, the systematic comparison between RGB-only and chlorophyll-sensitive indices within the same YOLO architecture has shown a 34% relative F1-score improvement attributable solely to spectral-index selection, and the use of CARI-based representations outperforms the commonly used red-edge and NDVI features for early-stage Botrytis detection under operational vineyard conditions. Finally, the determination of an end-to-end pipeline producing geolocatable infection hotspots, enabling actionable precision interventions rather than plot-level risk maps.

Therefore, the investigation of the chlorophyll-absorption-based vegetation indices (CARI/MCARI) provide statistically and operationally superior features for UAV-based object detection of *Botrytis cinerea* compared to RGB imagery. So, as we will see, CARI-based inputs significantly improve detection precision and recall compared to RGB under field conditions.

## 2. Materials and Methods

### 2.1. Study Overview

In this study an AI-UAV based detection system has been developed to detect *Botrytis cinerea* micoinfection in vineyards. The software of this system integrates multispectral imaging (taken with UAVs), computation of vegetation indices, and deep learning to detect Botrytis micoinfections in vines. The methodology used here agrees with the Agriculture 5.0 paradigm in which we have a combination of automated aerial sensing with artificial intelligence to enable data-driven decision-making and early detection of crop stress.

### 2.2. Study Area and Data Acquisition

The dataset used in this work originates from the experimental vineyard in Villena (Spain) located at the coordinates (lat, long) = (38.624217, −0.955320). Multispectral images were collected using the multispectral camera of a DJI Mavic 3M (DJI, Nanshan District, Shenzhen, China). While limited geographically, the physiological basis of CARI makes the approach transferable across grape varieties and canopy architectures. The UAV flights were conducted under stable illumination and low-wind conditions at an altitude of 40 m above ground level, ensuring a ground sampling distance (GSD) of approximately 1.1 cm/pixel for 20MP RGB camera (for 5MP multispectral camera is around 4.2 cm/pixel). Each flight captured five spectral bands—Green (560 nm), Red (650 nm), Red Edge (730 nm), and Near-Infrared (860 nm)—enabling the computation of chlorophyll sensitive vegetation indices. Radiometric calibration was performed using MicaSense calibration panels to correct for irradiance variations following the procedures described by Fawcett et al. [3].

### 2.3. Image Pre-Processing and Vegetation Indices

RAW images were processed in Python 3.11 using the MicaSense SDK (https://github.com/micasense/imageprocessing accessed on 4 January 2026) and standard libraries such as OpenCV (v4.10.0.84) [14], Rasterio (v1.3.10) [15], NumPy (v2.0) [16], and Matplotlib (v3.8.4) [17]. Band alignment was performed using the Scale-Invariant Feature Transform (SIFT) algorithm to ensure accurate pixel correspondence between spectral channels [18]. The reflectance values were normalized and a series of vegetation indices were computed, including CIG, CIRE, CVI, NDRE, NDVI, GNDVI, NGRDI, CARI, and MCARI. These indices were selected for their sensitivity to chlorophyll content, foliar pigmentation, and physiological stress, as supported by Haboudane et al. (2004) [1] and Main et al. (2011) [2].

In our case, the drone (DJI Mavic 3M) captures high-resolution images across multiple spectral bands: Green (560 nm ± 16 nm), Red (650 nm ± 16 nm), Red Edge (730 nm ± 16 nm), Near-Infrared (860 nm ± 26 nm) at 5MP, and standard RGB at 20MP. Each capture results in four images (one per band), which are transmitted to a cloud in TIFF format to preserve image quality and spectral information and another one in JPEG format containing RGB information.

The following tasks are needed for each capture:

- Metadata Extraction: Extracts geolocation, camera settings, and other relevant metadata from the images.
- Vignetting Correction: Applies correction algorithms to mitigate vignetting effects present in the images.
- Image Alignment: Uses a transformation matrix to align images from different spectral bands, correcting distortions due to multiple cameras.
- Spectral Indices Calculation: Computes nine spectral indices based on the different spectral bands to analyse vegetation health and characteristics computed from the captured spectral bands Near-Infrared (NIR), Red (R), Red Edge (RE), and Green (G). Each index provides insights based on specific combinations of these bands. Table 2 lists the indices, their corresponding formulas, and full names.

**Figure 3:**
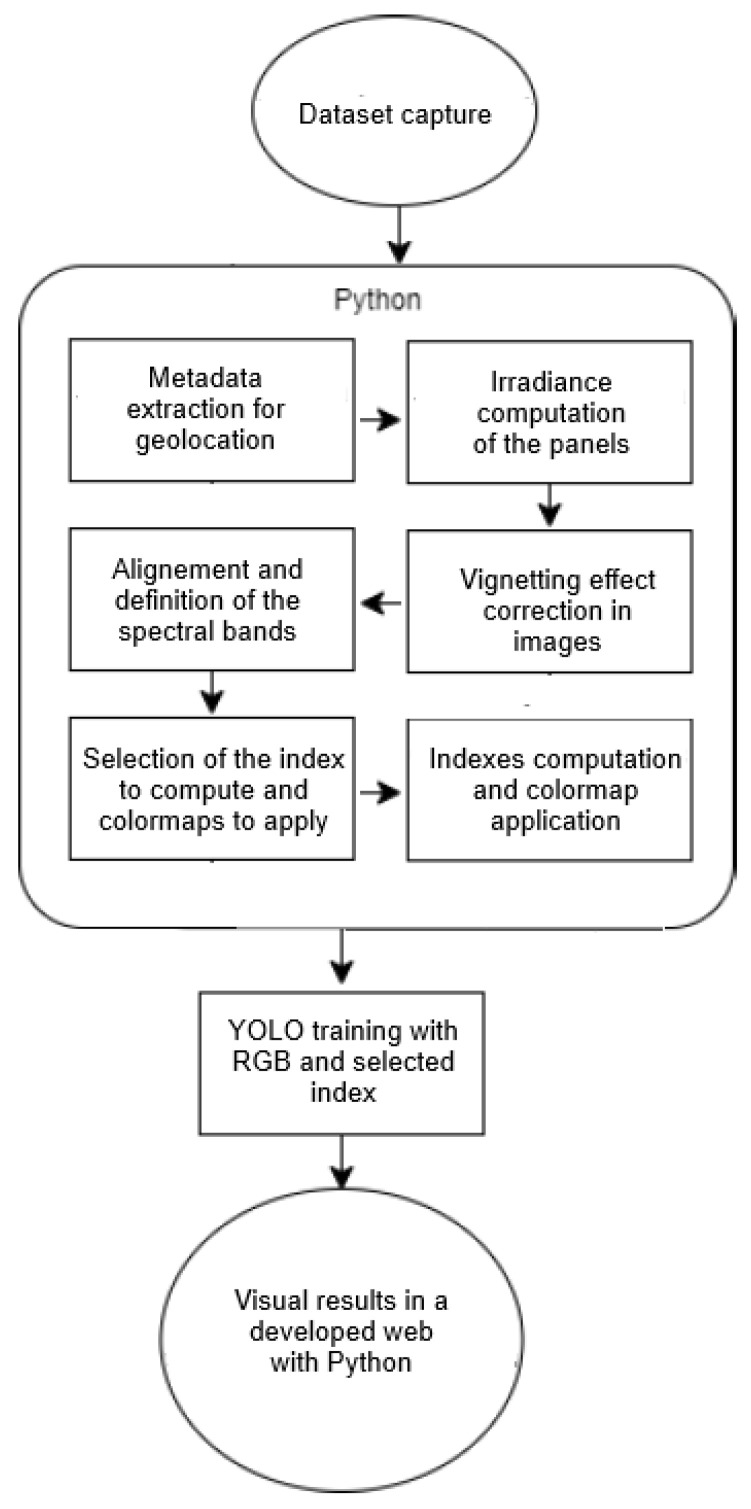
Flux diagram of the detection process.

Figure 3 shows in two parts the flux diagram of the image processing and the detection process with YOLO.

Table 2 describes the different multispectral indexes that have been considered to be computed in the disease detection monitoring system. In this table, R stands for ‘Red reflectance’, G for ‘Green reflectance’. RE for ‘Red Edge reflectance’, and NIR for ‘Near Infrared reflectance’.

### 2.4. Dataset Preparation and Annotation

The image corpus used in this study was acquired using a DJI Mavic 3 Multispectral UAV over an experimental vineyard located in Villena (Spain). Data collection was conducted during the growing season under stable illumination and low-wind conditions. The multispectral payload of the UAV captures five spectral bands—Green (560 nm), Red (650 nm), Red Edge (730 nm), and Near-Infrared (860 nm) at 5 MP resolution, as well as RGB imagery at 20 MP resolution.

A total of 1575 RGB images were collected, and the corresponding multispectral image sets each consisted of four aligned spectral band images. The resulting ground sampling distance was approximately 1 cm/pixel for RGB images and 4 cm/pixel for multispectral images, allowing individual grape clusters and leaf regions to be resolved. Images were radiometrically calibrated using MicaSense calibration panels and geometrically aligned prior to analysis.

The dataset includes images of both healthy vegetation and vines affected by Botrytis cinerea, with infection symptoms verified through field inspection. Manual annotation was performed using the Roboflow platform [20] by domain-trained operators. Bounding boxes were assigned to image regions exhibiting visible or spectral indicators of *Botrytis cinerea* infection, while healthy vegetation areas were left unlabelled, consistent with single-class object detection protocols.

In total, a number of approximately 200 annotated instances of *Botrytis cinerea* were identified across the dataset. The annotated corpus was divided into training (70%), validation (20%), and testing (10%) subsets at the image level to avoid data leakage. Data augmentation techniques, including rotation, scaling, brightness adjustment, and cropping, were applied exclusively to the training set to improve generalization, consistent with methods in plant-disease detection literature [4].

This dataset provides a representative sample of vineyard conditions, canopy densities, and illumination variability, supporting the evaluation of the proposed UAV–AI detection framework under realistic operational scenarios.

### 2.5. Deep Learning Architecture

For automatic detection, the You Only Look Once (YOLOv8) architecture was used, a state-of-the-art object detection network known for real-time performance [8]. The model was trained for 300 epochs using a learning rate of 0.001, stochastic gradient descent (SGD) optimization, and batch normalization. Transfer learning was applied using pre-trained weights from the COCO dataset to accelerate convergence. All experiments were executed on a workstation equipped with an AMD Ryzen 5 3600 CPU, 16 GB RAM, and an NVIDIA RTX 3060 GPU (12 GB VRAM). The evaluation of the model was based on the precision, recall, and F1-score metrics.

### 2.6. Web-Based Visualization

A lightweight web application was developed using the Flask framework [21] and Flask-SQLAlchemy for database management. The platform allows users to visualize both raw RGB images and processed vegetation index layers, along with YOLO-based detection overlays. Public users can access limited visualizations, while registered users can query complete datasets for advanced analysis.

### 2.7. Performance Evaluation

In order to evaluate the model performance, and considering confusion matrix with true positive (TP), true negative (TN), false positive (FP) and false negative, we have used Precision, Recall, and F1-score metrics, as defined in Equations (1)–(4). Confusion matrices and precision–recall curves were computed to assess classification robustness.

(1) $$\begin{array}{cc} \mathrm{Accuracy} & =\frac{TP+TN}{TP+TN+FP+FN} \end{array}$$

(2) $$\begin{array}{cc} \mathrm{Precision} & =\frac{TP}{TP+FP} \end{array}$$

(3) $$\begin{array}{cc} \mathrm{Recall} & =\frac{TP}{TP+FN} \end{array}$$

(4) $$\begin{array}{cc} F1-score & =\frac{2\cdot \mathrm{Precision}\cdot \mathrm{Recall}}{\mathrm{Precision}+\mathrm{Recall}} \end{array}$$

To this end, we have evaluated and compared two training configurations: one using RGB images and another using the CARI index (Chlorophyll Absorption Ratio Index). The process for calculating Average Precision consists of several steps. First, the model is used to generate the prediction scores. The model outputs bounding box predictions associated with confidence scores. These predictions are ranked by confidence, and a precision–recall curve is computed by varying the detection threshold. The Average Precision (AP) is then calculated as the area under the precision–recall curve. The mean Average Precision (mAP) is obtained by averaging AP values across all classes, following standard object detection evaluation protocols.

The mean Average Precision (mAP) is computed by calculating the AP for each class and then taking the average across all classes, as shown in Equation (5). Because mAP captures the balance between precision and recall and accounts for both false positives and false negatives, it is considered an appropriate metric for most detection applications.

(5) $$mAP=\frac{1}{N}\sum_{i=1}^{N}AP_{i}$$

## 3. Results and Discussion

### 3.1. Model Performance

This study developed a UAV-AI–based detection system designed to identify *Botrytis cinerea* infections in vineyard crops, integrating multispectral imaging, vegetation indices, and deep learning.

**Figure 4:**
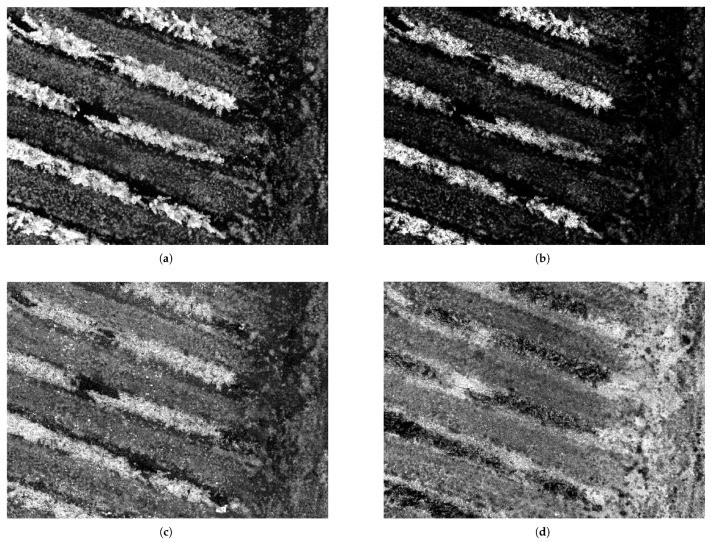
Multispectral indexes combinations: (**a**) CARI, (**b**) MCARI, (**c**) CIG, and (**d**) CIRE.

**Figure 5:**
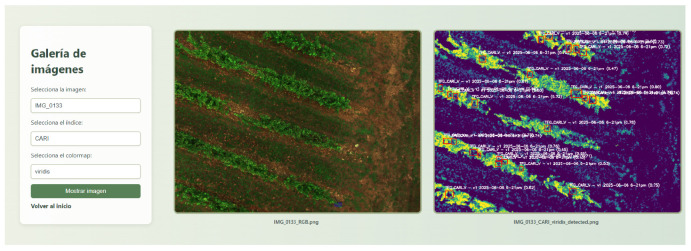
Detection results of Botrytis with YOLO-CARI model.

The developed UAV-AI detection system has demonstrated strong performance in identifying *Botrytis cinerea* symptoms under real vineyard conditions. Table 3 shows a summary of the performance evaluation for each dataset. The quantitative performance metrics reported in Table 3 were calculated throughout the test subset, comprising approximately 200 annotated *Botrytis cinerea* instances across 1575 multispectral images. Each detected bounding box was evaluated against ground-truth annotations generated on the Roboflow platform. Figure 4 and Figure 5 present representative examples selected from this evaluation to illustrate typical spectral responses and detection outputs.

The superior performance of the CARI-based configuration can be explained by the different spectral sensitivities of the vegetation indices and their relationship with chlorophyll absorption mechanisms. NDVI, defined as the normalized difference between near-infrared (NIR) and red reflectance, combines chlorophyll absorption effects in the red band with structural scattering effects in the NIR band. While NDVI is effective for assessing vegetation vigor and biomass, it tends to saturate in dense canopies and is strongly influenced by canopy geometry, row structure, soil background, and illumination variability, which limits its sensitivity to early physiological stress and pathogen-induced pigment degradation [1,2].

The Chlorophyll Index Green (CIG) improves sensitivity to chlorophyll concentration by incorporating green reflectance, which is inversely related to pigment content. However, green-band reflectance is only indirectly linked to chlorophyll absorption and remains sensitive to changes in illumination, leaf angle distribution, and specular reflection, particularly under heterogeneous vineyard conditions. Moreover, the continued reliance on NIR reflectance introduces structural effects that are not directly related to disease progression.

In contrast, the Chlorophyll Absorption Ratio Index (CARI) is specifically designed to isolate chlorophyll absorption features by exploiting the spectral contrast between the red and red-edge bands while applying a baseline correction using green reflectance. This formulation reduces the influence of non-physiological brightness variations, soil background effects, and canopy structural heterogeneity, allowing CARI to more effectively capture subtle biochemical changes associated with chlorophyll degradation [1]. Red-edge-based indices have been shown to be particularly sensitive to early stress conditions, as shifts in the red-edge slope occur prior to visible symptoms and before significant changes in NIR reflectance are observed [2,22]. Figure 4 shows the multispectral combinations of images for these indexes.

This property is especially relevant for the detection of *Botrytis cinerea*, as early infection stages primarily affect chlorophyll content and cellular integrity rather than canopy structure. Consequently, indices that emphasize chlorophyll absorption dynamics rather than structural contrast are better suited for early disease detection. The enhanced physiological specificity of CARI results in higher local contrast and reduced background variability in multispectral representations, which in turn facilitates more discriminative feature extraction by the YOLO-based object detection model.

The YOLOv8 model trained on multispectral data derived from the CARI (Chlorophyll Absorption Ratio Index) achieved a precision of 0.93, recall of 0.90, and F1-score of 0.91. In contrast, the RGB-based model yielded precision and recall values of 0.72 and 0.65, respectively, corresponding to an F1-score of 0.68. The 34% relative increase in F1 confirms the added value of spectral indices in enhancing feature separability and robustness to illumination changes. Similar results were reported by Kerkech et al. (2020) [4] and Vélez et al. (2023) [7], who observed improved classification accuracy using multispectral UAV data for vine disease detection.

### 3.2. Model Robustness and Environmental Conditions

The system maintained reliable detection across varying sunlight and canopy densities, with an average inference time below 50 ms per image—suitable for near real-time UAV operation. However, accuracy slightly declined under overcast conditions, consistent with the limitations reported by Fawcett et al. (2020) [3]. The trained YOLO-CARI model’s resilience suggests that integrating reflectance calibration and adaptive exposure correction can further enhance operational stability. Figure 5 shows an inference for the detection of *Botrytis* in a field in Villena with the YOLO-CARI model.

### 3.3. Comparison with Related Studies

Compared with existing approaches, such as pixel-based classification [23] or CNN models trained solely on RGB imagery [24], the proposed method achieves a higher detection precision with lower computational overhead. Furthermore, the web-based interface enables end-users to visualize detections and spectral indices, bridging the gap between AI outputs and actionable agricultural decisions—an essential component of precision agriculture systems [25,26].

### 3.4. Implications for Agriculture 5.0

The presented UAV-AI configuration illustrates how autonomous sensing and intelligent data analytics can contribute to the transition from Agriculture 4.0 to Agriculture 5.0. By coupling machine vision with automated aerial platforms, the system supports continuous monitoring, early disease mitigation, and resource-efficient interventions. This integration exemplifies the principles of smart, sustainable, and self-adaptive agricultural ecosystems envisioned for the next generation of precision farming [9,27].

## 4. Conclusions

This study presents an integrated UAV–AI system for early detection of *Botrytis cinerea* in vineyard crops, demonstrating how multispectral imaging and deep learning can be effectively combined to advance Agriculture 5.0 principles. The proposed system, based on the YOLOv8 architecture and CARI vegetation index, achieved an F1-score of 0.91, outperforming the RGB-based configuration by approximately 34%. This improvement confirms that chlorophyll-sensitive spectral indices and red-edge reflectance provide superior discrimination of stress-related patterns, enabling early identification of fungal infections.

The developed framework also highlights the potential to combine real-time UAV sensing with autonomous AI analytics for operational decision support. The inference speed achieved (<50 ms per image) and the consistent accuracy under field conditions position the system as a practical tool for precision viticulture, capable of reducing manual inspection time and improving sustainability through targeted interventions.

Beyond viticulture, this UAV–AI configuration can be adapted to a wide range of crops and stress factors, contributing to the transition from reactive to predictive agricultural management. Future work will focus on integrating temporal monitoring, adaptive learning, and edge-computing capabilities to enhance autonomy and scalability, further aligning with the Agriculture 5.0 paradigm of intelligent, data-driven, and environmentally responsible farming systems.

## Figures and Tables

**Table 1:** Summary of the contributions in the state of the art.

Study	What It Does	What It Cannot Do	What You Add
Pinheiro et al. [6]	RGB YOLO lesion detection	No early-stage detection; no multispectral	Early-stage spectral sensitivity
Kerkech et al. [4]	Multispectral segmentation	Pixel-level, not object-level; no geolocation	Object detection + hotspots
Vélez et al. [7]	Risk modeling	No direct detection	Direct pathogen detection

**Table 2:** List of Computed Spectral Indices [19].

Index	Full Name	Formula
CIG	Chlorophyll Index Green	$\frac{\mathrm{NIR}}{G}-1$
CIRE	Chlorophyll Index Red Edge	$\frac{\mathrm{NIR}}{\mathrm{RE}}-1$
CVI	Chlorophyll Vegetation Index	$\frac{\mathrm{NIR}\times R}{G^{2}}$
GNDVI	Green Normalized Difference Vegetation Index	$\frac{\mathrm{NIR}-G}{\mathrm{NIR}+G}$
NDRE	Normalized Difference Red Edge	$\frac{\mathrm{NIR}-\mathrm{RE}}{\mathrm{NIR}+\mathrm{RE}}$
NDVI	Normalized Difference Vegetation Index	$\frac{\mathrm{NIR}-R}{\mathrm{NIR}+R}$
NGRDI	Normalized Green Red Difference Index	$\frac{G-R}{G+R}$
CARI	Chlorophyll Absorption Ratio Index	RE−R−0.2×(RE−G))
MCARI	Modified Chlorophyll Absorption Ratio Index	$\mathrm{RE}-R-0.2\times \left(\mathrm{RE}-G\right)\times \frac{\mathrm{RE}}{R}$

**Table 3:** Performance evaluation of the action of the YOLO training with different datasets.

YOLO Training	mAP@50	Precision	Recall	F1-Score
RGB	68.5%	71.9%	64.8%	68.1%
NDVI	78.2%	80.1%	73.6%	76.7%
CIG	85.4%	87.2%	81.5%	84.2%
CARI	93.9%	92.6%	89.6%	91.1%

## Data Availability

The raw data supporting the conclusions of this article will be made available by the authors on request.
